# Exploration of HER2 (ERBB2) immunohistochemistry in non-small cell lung cancer: correlation with ERBB2 mutational status: experimental research

**DOI:** 10.1097/MS9.0000000000000719

**Published:** 2023-05-03

**Authors:** Anthony B. Cardillo, Sierra Kovar, Nitin Roper, David G. Hicks, Moises J. Velez

**Affiliations:** a Department of Pathology and Laboratory Medicine, University of Rochester, Rochester, NY; b Developmental Therapeutic Branch, Center for Cancer Research, NCI, NIH, Bethesda, MD

**Keywords:** ERBB2, HER2, immunohistochemistry, non-small cell lung cancer

## Abstract

*ERBB2* (*HER2*) is a gene in humans that encodes the ERBB2 protein, a member of the epidermal growth factor receptor family. Non-small cell lung carcinomas do not commonly harbour *ERBB2* mutations, with clinical trials conducted to assess for targeted response and progression-free survival. We retrieved cases of lung adenocarcinoma with next-generation sequencing proven *ERBB2* point mutations (*n*=8) or amplifications (*n*=11) and assessed the concordance of commercially available ERBB2 (HER2) immunohistochemical antibodies with the next-generation sequencing result. At present, no commercially available ERBB2 clone can accurately detect *ERBB2* mutations consistently in non-small cell lung carcinoma specimens, but amplifications can be detected with reasonable diagnostic accuracy.

## Introduction

Highlights

- Non-small cell lung carcinoma (NSCLC) cases with next-generation sequencing proven *ERBB2* point mutations (*n*=8) or amplifications (*n*=11) were assessed with commercially available *ERBB2* (*HER*2) immunohistochemical antibodies.
- Our study supports that, at this time, none of the commercially available ready-to-use *ERBB2* immunohistochemical clones are suitable for mutation screening in NSCLC when using established grading systems or using the presence of staining alone.
- Reasonable diagnostic accuracy can be obtained to identify HER2-amplified NSCLC specimens with 3+ *HER2* protein expression.

*ERBB2* is a gene in humans that encodes the ERBB2 protein, a member of the epidermal growth factor receptor family^1^. *ERBB2*, also known as *HER2* or HER2/neu, is an oncogene^2^ perhaps best known for its characterization and sensitivity to trastuzumab^3^ and lapatinib^4^ in breast cancer. Approximately 3% of non-small cell lung carcinomas (NSCLCs) harbour *ERBB2* mutations^1^, with clinical trials conducted to assess for targeted response and progression-free survival.

In 2004, Stephens *et al* ^5^. reported ~10% of adenocarcinomas harbour an *ERBB2* mutation in the kinase domain. Arcila et al further found that *HER2* mutations identify a distinct subset of lung adenocarcinomas with an estimated incidence rate between 1000 and 2000 patients in the United States each year^6^. It is now estimated that *HER2* mutations represent 1.7% of all NSCLC cases and have higher proportions of never-smokers^7^ compared with other mutations.

One of the first trials^8^ targeting *HER2* aberrations showed partial responses to dacomitinib (an EGFR-family multi-kinase receptor inhibitor) but the response was limited to those patients with *ERBB2* exon 20 insertions and not amplifications. Another phase II study, the CUSTOM trial, attempted to determine efficacy of lapatinib, a HER2 tyrosine kinase inhibitor best known as a treatment for HER2-positive breast cancer, on patients with HER2 mutations. Unfortunately, statistically significant analysis could not be performed with the trial design due to the inherently low incidence of HER2 aberrancies^9^. The SUMMIT^10^ trial in 2018 found that neratinib prolonged median progression-free survival rate in a cohort of 26 NSCLC patients with HER2 mutations, but only one patient achieved a response that meets the RECIST criteria^11^.

Pyrotinib, a pan-ERBB tyrosine kinase inhibitor, was found to have an objective response rate (determined by an independent committee using RECIST) of 30% in patients with advanced NSCLC who had previously received platinum-based chemotherapy^12^, demonstrating potential utility in screening for *HER2* mutations in NSCLC of all stages.

Following numerous phase II clinical trials, the first antibody-drug conjugate to be tested in lung tumours, ado-trastuzumab emtansine, demonstrated high partial response rates (44%) to tumours harbouring ERBB2 mutations^13^. This conjugate therapy was closely followed by trastuzumab deruxtecan in the trial DESTINY-Lung01^14^, which displayed efficacy in 55% of HER2-mutant NSCLCs.

Given the pipeline of targeted *HER2* therapies for lung cancer, the need for widely available screening tests is fast increasing. We sought to characterize three well known HER2 immunohistochemical (IHC) clones in the detection of ERBB2 protein expression in NSCLC with known *ERBB2* alterations, detected by next-generation sequencing (NGS). IHC expression was graded using the Breast and Gastric^15^ HER2 scoring systems to determine correlation with underlying molecular alterations. We assessed for cross-reactivity among additional molecular subtypes, including *BRAF*, *KRAS*, *MET*, *NRAS*, and *EGFR*, along with cross-reactivity to an extended panel of specimens without any known mutations.

## Materials and methods

### Specimen acquisition

A total of nineteen lung adenocarcinoma specimens, belonging to 19 individuals, were retrieved that met the following criteria: (1) a confirmed diagnosis of NSCLC by a thoracic pathologist, (2) had a mutation or amplification of the *ERBB2* gene detected by NGS, and (3) received molecular testing results prior to therapy. Subsequent molecular testing results following therapy were excluded. Eleven of the specimens were locally retrieved from our archives from eleven different patients; the remaining eight specimens were retrieved from a collaborating institution from five different patients. All specimens were analyzed within the two previous years. NGS data for the specimens were available through two separate testing platforms:

Mayo LNGPR (Mayo Medical Laboratories) is an NGS panel that tested for somatic alterations in 12 different genes. For ERBB2 specifically, copy number variations were not detected.

FoundationOne CDx (Foundation Medicine, Inc) is an NGS platform which interrogated 324 different genes for four types of genetic alterations: substitution, indels (insertion or deletion), copy number alterations, and rearrangements. Absolute copy number valuations were reported based on FoundationOne FISH concordance studies.

### Immunohistochemistry

HER2 protein expression was accessed with HercepTest IHC (Agilent) using the Dako Omnis platform; Ventana clone 4B5 rabbit monoclonal antibody (Roche) using the Benchmark Ultra platform; and Leica clone CB11 (Leica Biosystems) using the Leica Bond III platform. HercepTest and Ventana clone 4B5 are companion diagnostic assays for the detection of HER2 overexpression for treatment eligibility with transtuzumab and pertuzumab or ado-tratuzumab emansine, respectively, in HER2-positive breast and gastric cancers. All IHC clones used in this study are ready-to-use assays intended for in-vitro diagnostic use and were optimized using the provided recommended instructions, platforms and recommended reagents. Antibody concentrations were unaltered.

### Discriminatory ability to Stain *ERBB2*-mutants and *ERBB2*-amplifications

Eight of the specimens had *ERBB2* point, insertion, duplication, or deletion mutations and comprised the “mutation” cohort (Table 1). These mutations were detected using both the Mayo LNGPR (*n*=4) and FoundationOne CDx panels (*n*=4). One of the eight *ERBB2* mutations harboured a pathogenic *KRAS* co-mutation. The remaining 11 lung specimens were *HER2*-amplified by NGS or FISH; these 11 specimens comprise the “amplification” cohort. Amplifications detected by NGS (*n*=4) were only detected with FoundationOne CDx and showed a HER2 copy number of 6–28. Three *HER2* IHC clones were run on each specimen in parallel: dako (HercepTest™), Ventana (4B5), and Leica (CB11). Each clone was graded by both the breast and gastric scoring systems as described in protocols (2021) set forth by the College of American Pathologist (CAP). As control tissue, 22 (*n*=22) specimens without molecular alterations by NGS (“pan-negative”) were stained, along with 24 (*n*=24) specimens with a mutation in *BRAF* (*n*=4), *KRAS* (*n*=6), *MET* (*n*=5), *NRAS* (*n*=1), or *EGFR* (*n*=8). The *ERBB2* and *KRAS* co-mutation is excluded from the *KRAS* cohort (Table 1). All twenty-two specimens were used as negative controls in the mutation cohort, whereas only eight of the negative controls (8/24) were used for the amplification cohort—these eight used the FoundationOne panel that tests for the presence (or absence) of *ERBB2*-amplifications. These non-ERBB2-mutation specimens allow for an approximation of specificity and sensitivity in both cohorts. A total of 66 specimens (Table 1) were stained in triplicate with the aforementioned three clones. IHC correlation with mutational status was then assessed. To assess for HER2 antibody specificity in the detection of *HER2* mutations or amplification, we first determined if “any staining” was present; defined by nuclear, cytoplasmic or membranous staining of any intensity (1+, 2+, 3+).

**Table 1:** Numerical breakdown of specimens by presence or absence of specific mutations and *ERBB2-*mutation variants

Pan-negative specimens	**22**		
Negative by Mayo NGS	14		
Negative by FoundationOne	8		
Total mutants	**44**		
ERBB2-mutants	**20**		
Non-amplification mutations	8*		
Amplifications	11		
Non-ERBB2-mutants	**24**		
*KRAS*	6		
*MET*	5		
*BRAF*	4		
*NRAS*	1		
*EGFR*	8		
Platform	*ERBB2* non-amplification mutations (*n*=8)	Variant type	Pathogenic co-mutation
Mayo	ERBB2 exon 19 p.L755S (Leu755Ser)	Substitution—missense	KRAS p.G12D (Gly12Asp)
Mayo	ERBB2 exon 20 p.Y772_A775dup (Tyr772_Ala775dup)	Insertion—in frame	—
Foundation medicine	ERBB2 exon 20 (A775_G776insYVMA)	Insertion—in frame	—
Foundation medicine	ERBB2 exon 20 (A775_G776insYVMA)	Insertion—in frame	—
Foundation medicine	ERBB2 exon 17 (V659E)	Substitution—missense	—
Foundation medicine	ERBB2 exon 17 (G660D)	Substitution—missense	—
Foundation medicine	ERBB2 (K676M)	Substitution—missense	—
Foundation medicine	ERBB2 exon 20 (A775_G776insYVMA)	Insertion— in frame	—

NGS, next-generation sequencing.

### Breast versus gastric scoring system for NSCLC

In comparing the stains statistically, any amount of staining (i.e. a non-zero score) is used to improve sensitivity in detecting differences between the stains. Clinically, since no formal HER2 grading system for lung cancer exists, the stained slides were additionally assessed using the Breast (Table 2) and Gastric scoring systems (Table 3) for HER2 staining to determine if differences arise with either scoring system when applied to NSCLC. Three pathologists scored each stained slide by consensus, blinded to the NGS and FISH results of the specimen. The CAP breast HER2 scoring system is based on interpretation from the 2018 American Society of Clinical Oncology (ASCO)/CAP HER2 Guidelines^16^. The gastric scoring system is based on the 2016 recommendations for HER2 Testing and Clinical Decision Making in Gastroesophageal Adenocarcinoma, from CAP, the American Society of Clinical Pathology and ASCO^15,17^ which are currently used on biopsy and resection specimens.

**Table 2:** The Breast HER2 IHC scoring system; 2021 College of American Pathologist (CAP)

Result	HER2 IHC Interpretation
Negative (0)	No staining observed or membrane staining that is incomplete and is faint/barely perceptible and within ≤10% of tumour cells.
Negative (1+)	Incomplete membrane staining that is faint/barely perceptible and within >10% of tumour cells.
Equivocal (2+)	Weak to moderate complete membrane staining in >10% of tumour cells or complete membrane staining that is intense but within ≤10% of tumour cells.
Positive (3+)	Complete membrane staining that is intense and >10% of tumour cells.

IHC, immunohistochemical.

**Table 3:** The gastric HER2 IHC scoring system; 2021 College of American Pathologist (CAP)

Result	HER2 IHC Interpretation in Surgical Resections	HER2 IHC Interpretation in Biopsy Specimens
Negative (0)	No or lack of membranous staining in <10% tumour cells	No or lack of membranous staining in any tumour cells
Negative (1+)	Faint membranous staining in ≥10% of tumour cells; cells can show staining only in part of their membrane	Cluster of ≥5 tumour cells with faint perceptible membranous staining in any percentage of tumour cells
Equivocal (2+)	Weak to moderate complete, basolateral or lateral membranous staining in ≥10% of tumour cells	Cluster of ≥5 tumour cells with a weak to moderate complete, basolateral, or lateral membranous staining in any percentage of tumour cells
Positive (3+)	Strong complete, basolateral or lateral membranous staining in ≥10% of tumour cells	Cluster of ≥5 tumour cells with strong complete basolateral, or lateral membranous staining in any percentage of cancer cells

IHC, immunohistochemical.

### Statistical methods

IHC correlation with mutational status was assessed using Barnard’s test^18^, a statistically more powerful alternative to Fischer’s exact test for 2×2 contingency tables, with a significance level of 0.05. Statistics were performed using the Python programming language. CIs (95%) were obtained using Wilson’s method^19^.

## Results

### Mutation cohort

#### Discriminatory ability to Stain *ERBB2*-mutants

**Figure 1:**
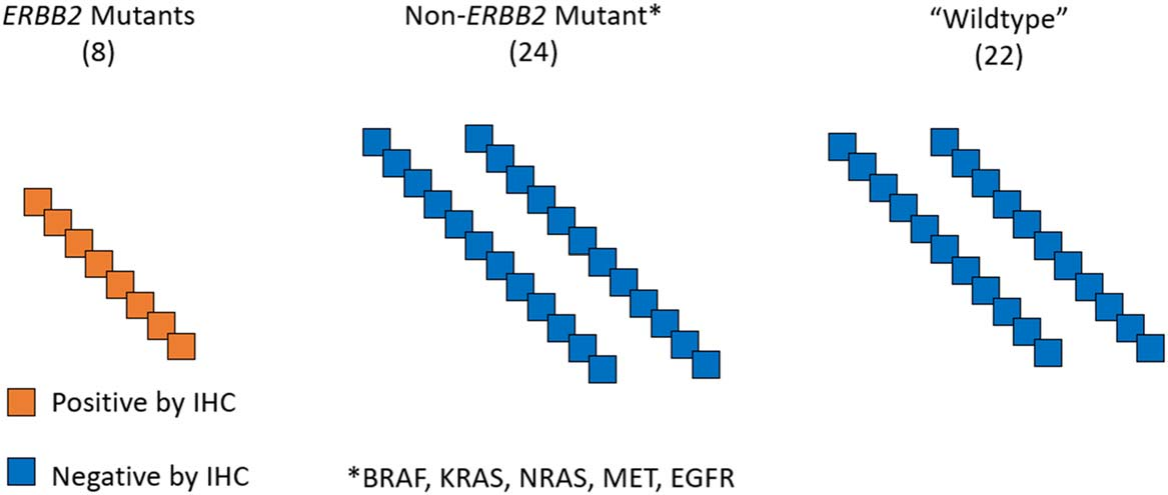
An ideal visual representation of 100% IHC concordance within our testing design. Slides are definitively sorted into three categories by their mutation status per NGS. A perfect ERBB2 IHC stain would only positively stain slides containing the *ERBB2*-mutated specimens (left), while not staining slides with other mutations (middle) or slides with no known mutation (right). IHC, immunohistochemical; NGS, next-generation sequencing.

The clones were first assessed for the presence of “any staining” in at least 1% of the tumour cell population, where “any staining” refers to a non-zero score that may represent detection of expression. Figure 1 demonstrates a perfect IHC test.

**Figure 2:**
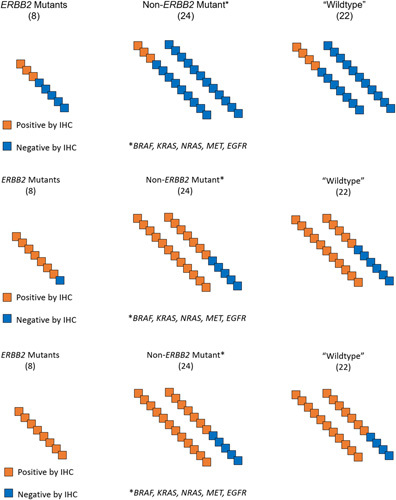
Visual representation of concordance between three *HER2* IHC assays. (Top Panel) Dako HercepTest clone performance, with a sensitivity of 38% and specificity of 84%. (Middle Panel) Ventana (4B5) clone performance, with a sensitivity of 88% and specificity of 26%. (Bottom Panel) Leica (CB11) clone performance, with a sensitivity of 100% and specificity of 20%. IHC, immunohistochemical.

HercepTest showed any degree of staining in 3/8 of *ERBB2* mutation specimens, for a sensitivity of 38% (95% CI, 14–75%). Additionally, HercepTest reactivity in the presence of other mutations is as follows (confidence intervals omitted for brevity): *BRAF* (0/4, 0%), *KRAS* (2/6, 33%), *MET* (2/5, 40%), *NRAS* (0/1, 0%), and *EGFR* (0/8, 0%). It showed any degree of staining in 3/21 of pan-negative specimens (one specimen did not have an adequate control). Combined with the cross-reactivity data, the final specificity is 38/45, or 84% (95% CI, 71–92%). The results are summarized in Figure 2.

The Ventana (4B5) clone showed any degree of staining in 7/8 of *ERBB2* mutation specimens, for a sensitivity of 88% (95% CI, 53–98%). Additionally, the 4B5 clone’s reactivity for other mutations is as follows (confidence intervals omitted for brevity): *BRAF* (3/4, 75%), *KRAS* (4/6, 67%), *MET* (4/5, 80%), *NRAS* (1/1, 100%), and *EGFR* (5/8, 63%). It showed any degree of staining in 15/19 of pan-negative specimens (three specimens did not have an adequate control). Combined with the cross-reactivity data, the final specificity is 11/43, or 26% (95% CI, 15–40%). The results are summarized in Figure 2.

The Leica (CB11) clone showed any degree of staining in 8/8 of *ERBB2*-mutation specimens, for a sensitivity of 100% (95% CI, 68–100%). Additionally, the CB11 clone reactivity for other mutations is as follows (confidence intervals omitted for brevity): *BRAF* (4/4, 100%), *KRAS* (5/6, 83%), *MET* (4/5, 80%), *NRAS* (1/1, 100%), and *EGFR* (5/8, 63%). It showed any degree of staining in 17/21 of pan-negative specimens (one specimen did not have an adequate control). Combined with the cross-reactivity data, the final specificity is 9/45, or 20% (95% CI, 11–34%). The results are summarized in Figure 2.

The previous data are summarized in Table 4 below, along with likelihood ratios if the tests were to be used in diagnostic practice:

**Table 4:** A tabular comparison of the basic statistical parameters for the three *HER2* IHC assays

	Dako	Ventana	Leica
Sensitivity (95% CI)	38 (14–75)	88 (53–98)	100 (68–100)
Specificity (95% CI)	84 (71–92)	26 (15–40)	20 (11–34)
Likelihood ratio, positive (95% CI)	2.4 (0.78–7.4)	1.2 (0.86–1.6)	1.3 (0.34–1.5)
Likelihood ratio, negative (95% CI)	0.7 (0.41–1.28)	0.5 (0.07–3.3)	0.0 (0.0–4.2)

None of the tests have a positive or negative likelihood ratio that excludes unity, implying a non-informative test within the confines of this study.

IHC, immunohistochemical.

**Figure 3:**
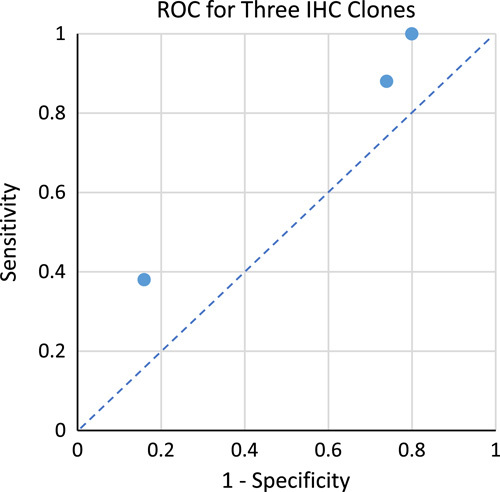
Receiver Operating Characteristic (ROC) for Three *HER2 IHC* assays. The sensitivity and false positive rate (1 – specificity) of each commercial IHC clone were plotted in ROC space. All three clones were similarly distanced from the line of no discrimination (blue dotted line) which implies no superior performance compared to random guessing. A perfectly informative test would lie at coordinates (0,1). IHC, immunohistochemical.

Viewing the tests in receiver operating characteristic space (Fig. 3) is a visual method for comparing the sensitivity-specificity tradeoff of binary-result tests. Graphically represented, a perfect test has 100% sensitivity and 100% specificity, and would lie in the top-left corner of receiver operating characteristic space. None of the commercial clones were significantly distant from the line-of-no-discrimination, the line achieved from random guessing of a binary outcome. This implies that the result of the test is not significantly informative.

#### Breast versus gastric scoring system for NSCLC

HercepTest did not correctly classify any *ERBB2*-mutation specimens as equivocal or positive. It classified all pan-negative specimens as negative using both systems. Thus, the statistical accuracy of the HercepTest clone is 18/29, or 62% (95% CI, 44–77%) in both scoring systems (Table 5).

**Table 5:** Comparison of breast and gastric scoring systems for *HER2* IHC staining.

	Dako	Ventana	Leica
Accuracy, breast system (95% CI)	62 (44–77)	64 (45–70)	69 (51–84)
Accuracy, gastric system (95% CI)	62 (44–77)	60 (41–77)	62 (44–77)

No clone had a clinically suitable accuracy for use as a screening or prognostic test for ERBB2 mutations.

IHC, immunohistochemical.

The 4B5 clone also did not correctly classify any *ERBB2*-mutation specimens as equivocal or positive, and incorrectly called two pan-negative specimens as positive by the Gastric scoring system. The statistical accuracy of the 4B5 clone for the gastric scoring system is 15/25, or 60% (95% CI, 41–77%). The accuracy for the Breast scoring system is 16/25, or 64% (95% CI, 45–70%). Results are summarized in Table 5.

The CB11 clone classified four *ERBB2*-mutation specimens as equivocal by both the Breast and GI criteria, and incorrectly called seven pan-negative specimens equivocal or positive by the Gastric scoring system and five pan-negative specimens equivocal or positive by the Breast scoring system. The statistical accuracy of the Leica clone for the Gastric scoring system is 18/29, or 62% (95% CI, 44–77%). The accuracy for the Breast scoring system is 20/29, or 69% (95% CI, 51–84%). Results are summarized in Table 5.

### Amplification cohort

All three IHC clones stained (1+ to 3+) ten of the eleven (10/11) available *ERBB2*-amplification specimens, for a sensitivity of 91%. These ten cases all displayed complete membranous and lateral or basolateral staining. For each IHC clone, one *ERBB2*-amplified (FISH proven) specimen was falsely negative (0+) for protein expression (Table 6).

**Table 6:** Breakdown of number of specimens per stain score by *HER2* IHC assays on proven *ERBB2*-amplified tissue.

Stain score	Dako	Ventana	Leica
0+	1	1	1
1+	2	2	2
2+	5	5	5
3+	2	2	2

Each stain was 100% concordant after blinded pathologist scoring; in other words, the resulting score was the same for each specimen no matter which clone was used.

IHC, immunohistochemical.

Of the eight specimens that were pan-negative by the more extensive Foundation panel, the Dako clone showed membranous staining (1+ to 3+) in zero specimens, the Ventana clone showed membranous staining in five specimens, and the Leica clone showed membranous staining in one specimen. The specificity for each is 100%, 38%, and 88%, respectively (Table 7).

**Table 7:** A tabular comparison of the basic statistical parameters for the three *HER2 IHC* assays when used on the amplification cohort.

	Dako	Ventana	Leica
Sensitivity (95% CI)	73 (39–94)	73 (39–94)	73 (39–94)
Specificity (95% CI)	100 (63–100)	38 (9–76)	88 (47–99)
Accuracy % (95% CI)	84 (62–94)	58 (36–80)	79 (57–91)
Likelihood Ratio, Positive (95% CI)	∞	1.2 (0.6–2.2)	5.8 (0.9–38)
Likelihood Ratio, Negative (95% CI)	0.3 (0.1–0.7)	0.7 (0.2–2.7)	0.3 (0.1–0.9)

Two of the tests (Dako HercepTest™ and Leica CB11) have likelihood ratios that exclude unity, implying an informative test within the confines of this study. Ventana 4B5 does not exclude unity for either likelihood ratio, which implies the test is not sufficient in this study to rule-in or rule-out amplification at a significance level of 0.05.

IHC, immunohistochemical.

Two of the tests (Dako HercepTest and Leica CB11) have a positive and negative likelihood ratio that excludes unity, implying an informative test within the confines of this study. Ventana 4B5 only excludes unity for the negative likelihood ratio, which implies a negative test is not sufficient to rule-out a disease at a significance level of 0.05.

## Discussion

Immunohistochemistry is a cheaper alternative for screening for mutations than NGS. With the increasing role of targeted therapies against *HER2*, including clinical trials ongoing in lung, an ERBB2 IHC stain with the proper sensitivity and specificity could potentially play a useful role in future workflows before reflexing to NGS or where NGS is not accessible. In the same way that detection of ERBB2-positivity by IHC or FISH modifies treatment in breast and gastric cancer, a similar adjuvant treatment may exist in the future for non-small cell lung cancers. Furthermore, IHC staining is lower complexity and more cost effective^20^ than NGS. Using the Breast Scoring System, which was equivalent or better than the Gastric system amongst all three clones, the accuracy of IHC tests ranged from 62% (Dako HercepTest) to 69% (Leica CB11). Ventana 4B5 showed in-the-middle accuracy of 64% in the ability to detect mutations. Though the accuracy of at least one of the IHC clones approaches 70%, this is likely due to the class imbalance between negative controls and ERBB2 positive specimens—thus, one would expect the positive predictive value of this stain to be lower in clinical practice. At present, the accuracy of the tests is likely not sufficient to achieve clinical utility. A possible cause for the lack of ERBB2 protein detection by IHC includes insufficient translation of the mutant protein leading to decreased or absent subcellular expression . Furthermore, the commercially available IHC clones were likely not produced to detect intracellular ERBB2 protein expression; with the focus on membranous expression given the increased number of transmembrane receptors following HER2 receptor activation and amplification. While cross-reactivity between transmembrane and intracellular ERBB2 protein is likely, the antigen binding sites for these IHC clones are not specified. It is also possible, localization of the antibody to antigenic sites may be hindered by mutation induced confirmation change.

Limitations of the study include a relatively low sample size. This is affected by both the number of NSCLC specimens that are sequenced via NGS, and the observed low percentage of NSCLC specimens that harbour ERBB2 mutations or amplifications. Future meta-analyses or studies with a greater sample size would increase the power of detecting a significant ability of these IHC clones to discriminate *ERBB2*-mutant specimens from wildtype specimens, should such ability exist.

In contrast, the DESTINY-Lung01^14^ trial conducted by Li et al included statistical results of their IHC methods. Using Ventana 4B5, the team stained 53 patient samples with known HER2 mutations. Forty-four patients had any amount of staining (83% sensitivity), which is statistically in-line with the 91% sensitivity observed in this study. Specificity was unable to be evaluated. The trial drug, trastuzaumab deruxtecan, showed efficacy even in patients for which no IHC staining was observed—this likely correlates to the false negatives that would be produced if IHC were used as a screening tool for *ERBB2* mutations. In the research setting, ERBB2 IHC should be carefully interpreted with respect to mutations due to its limited concordance.

## Conclusion

Our study supports that, at this time, none of the commercially available ready-to-use *ERBB2* IHC clones are suitable for mutation screening in NSCLC when using established grading systems or using the presence of staining alone. We were unable to detect or discriminate *ERBB2* point mutations consistently in NSCLC specimens. However, reasonable diagnostic accuracy can be obtained to identify HER2-amplified NSCLC specimens with 3+ *HER2* protein expression. In the setting of detecting amplifications, both the Dako HercepTest and Leica CB11 stains were able to discriminate between tissue containing amplifications versus negative controls, with 95% likelihood ratios that exclude unity. The 4B5 clone had a positive likelihood ratio excluding unity but did not possess negative discriminatory capability within a 95% CI This study suggests that, for the detection of *HER2* amplification (confirmed by either FISH or NGS), two commercially available clones may have sufficient sensitivity and specificity to warrant consideration as a screening test for *HER2*-amplified NSCLC, should such a treatment be found effective in NSCLC.

## Ethical approval

IRB (Institutional Review Board) approval was obtained.

## Consent

IRB waived.

## Source of funding

This project was internally funded by the department of Pathology and Laboratory Medicine at the University of Rochester Medical Center.

## Author contribution

All authors contributed and reviewed the manuscript for submission.

## Conflicts of interest disclosure

The authors declare no conflicts of interest.

## Disclosure

Advisory board Astra Zeneca and Diiachi Sankyo Speaker bureau AstraZeneca.

## Research registration unique identifying number (UIN)

NA.

## Guarantor

Moises J. Velez.

## Data availability statement

This data are not publically available.

## Provenance and Peer review

Not commissioned, externally peer-reviewed.
